# Improved outcomes in patients with positive metal sensitivity following revision total knee arthroplasty

**DOI:** 10.1186/s13018-019-1228-4

**Published:** 2019-06-17

**Authors:** Robert L. Zondervan, Jonathan J. Vaux, Michael J. Blackmer, Brett G. Brazier, Charles J. Taunt

**Affiliations:** 1 Michigan State University College of Osteopathic Medicine, 965 Wilson Rd, East Lansing, MI 48824 USA; 2 McLaren Orthopedic Hospital, 2727 S Pennsylvania Ave, Lansing, MI 48910 USA

**Keywords:** Metal allergy, Metal sensitivity, Arthroplasty, Orthopedics, Knee, Hip, Lymphocyte transformation test, LTT

## Abstract

**Background:**

Metal sensitivity as a cause for painful joint replacement has become increasingly prevalent; however, there is a lack of reported clinical outcome data from total knee arthroplasty patients with metal allergies. The purpose of this study was to determine whether patients presenting with a painful total knee arthroplasty with a positive metal sensitivity have improved outcomes following revision to a hypoallergenic implant.

**Methods:**

A retrospective review was conducted for patients that underwent a revision total knee arthroplasty after metal sensitivity testing over a 3-year period from January 1, 2015, to December 31, 2017. Based on the results of sensitivity testing, patients underwent revision total knee arthroplasty to a hypoallergenic component or a standard component. Following revision, patients returned to the clinic at an interval of 6 weeks, 5 months, and 12 months for functional, pain, and satisfaction assessment. Outcomes were compared within and between sensitivity groups.

**Results:**

Of the included patients, 78.3% (39/46) were positive for metal sensitivity. The most common metal sensitivity was to nickel (79.5%, 32/39). Both non-reactive and reactive patients significantly improved in range of motion after revision arthroplasty. The reactive group saw a 37.8% decrease in pain at 6 weeks post-revision (*p* < 0.001) Whereas, the non-reactive group only saw a moderate, non-significant improvement in pain reduction at 6 weeks post-revision (27.0%; *p* = 0.29). Frequency of pain experienced did not vary significantly between groups. Maximum metal lymphocyte transformation test (LTT) sensitivity score did not correlate with pain level at the time of revision (*R*^2^ = 0.02, *p* = 0.38) or percent improvement after revision (*R*^2^ = 0.001, *p* = 0.81). Overall, all patients reported being very satisfied after revision total knee arthroplasty; there was no difference between positive and negative sensitivity groups (*W* = 62, *p* = 0.89).

**Conclusions:**

Patients presenting with a painful knee arthroplasty and positive metal LTT have improved pain scores, walking function, and range of motion following revision to a hypoallergenic component. This study also provides a treatment algorithm for patients presenting with a painful knee replacement, in order to provide effective and timely diagnosis and management.

**Electronic supplementary material:**

The online version of this article (10.1186/s13018-019-1228-4) contains supplementary material, which is available to authorized users.

## Background

Primary knee arthroplasty is one of the most common orthopedic procedures performed each year in the USA. Due to the aging population, the number of total knee arthroplasty (TKA) procedures, and subsequently revision TKA, continue to rise [1]. Despite excellent results seen with primary knee arthroplasty, ranging from 75 to 92% good or excellent results, approximately one in five patients remain “dissatisfied” with their total joint replacement often leading to revision TKA [2, 3].

The most common causes leading to revision TKA reported in the literature include infection, instability, and malalignment [4]. Patients can present with a wide array of symptoms including pain, effusion, stiffness, and instability. Workup of the patient includes extensive labs and imaging, which leads to a diagnosis in the majority of patients. However, despite these measures, there remains a subset of patients with a painful TKA and a negative workup without an identifiable cause.

Recently, the idea of metal sensitivity as a cause for painful joint replacement has become increasingly prevalent. In general, 10–15% of the population demonstrate metal cutaneous sensitivity with the most prominent being nickel followed by cobalt and chromium [5–7]. The allergic reaction seen with metal sensitivity is a type IV T cell-mediated, or delayed type, hypersensitivity reaction [8]. The metal particles, or antigens, cross-react with proteins and form a complex, which subsequently triggers an immune response by the local tissues [9]. The metals most commonly found to cause cutaneous sensitivity are nickel, cobalt, and chromium, all of which are commonly found in the majority of TKA implants.

Multiple studies have shown that metal allergy in TKA exists and can be associated with a painful joint replacement and/or aseptic loosening. According to Hallab et al., the prevalence of metal allergy in patients with a well-functioning TKA is 25% and can be as high as 60% in those with a poorly functioning implant [7]. Additionally, patients with a failed implant had a twofold increased risk of metal allergy compared to those with a stable implant [7]. The clinical presentation of a patient with a painful TKA secondary to metal sensitivity initially presents with signs of decreased range of motion, pain at rest, and joint effusions [10]. While skin changes such as a rash are commonly seen with contact dermatitis, these types of changes are much less common as a presenting sign in TKA hypersensitivity due to the components residing deep within the tissues [11].

Traditionally, dermal patch testing has been the gold standard for detecting metal sensitivity reactions [12]. This consists of exposing the patient’s skin to a variety of antigens and observing for a delayed hypersensitivity-type reaction. However, interpretation of patch testing results can be subjective and unreliable [13, 14]. Patch testing does not allow for quantitative analysis and may not be appropriate for TKA hypersensitivity due to the difference in immunologic response differences between dermal contact and being embedded in tissue [15]. For these reasons, the literature does not currently support pre-testing of patients with a dermal patch test. Due to the lack of sensitivity with patch testing, the metal lymphocyte transformation test (LTT) has been advocated as a measure for metal sensitivity in total joint arthroplasty.

The LTT is an in vitro test that measures the proliferation of lymphocytes after exposure to tested antigens (metals). When compared to dermal patch testing, LTT has been found to have a greater sensitivity but a lower specificity [16]. In addition to having a higher sensitivity, the LTT is easily replicated and provides quantifiable results. In a study by Niki et al., they were able to effectively screen which patients would develop metal-related eczema following TKA using LTT [17]. However, the majority of allergic patients will not present with dermatological symptoms which underscores the need for a more sensitive test. Müller and Valentine-Thon studied hypersensitivity to titanium and demonstrated that of the 54 patients studied, none had a positive patch test, but 37.5% were positive using a LTT [18]. There are limitations to LTT, however, which include the previously mentioned lower specificity, the necessity for rapid transportation of blood samples secondary to rapid T-cell decay, and the relatively high out of pocket cost to patients [19]. Additionally, without clinical or histopathologic context, LTT results alone are likely insufficient for the diagnosis of immune-related TKA failure [20]. While there are reports of the use of LTT clinically, reported clinical outcome data from TKA patients with metal allergies is lacking. To address this need, the aim of our study was to determine whether patients presenting with a painful TKA and a positive LTT for metal sensitivity have improved outcomes following revision TKA to a hypoallergenic implant.

## Methods

A retrospective chart review was conducted in compliance with the Health Insurance Portability and Accountability Act (HIPAA) and with institutional review board (IRB) oversight. The study was deemed to be exempt from full IRB review. Charts from a single center were identified for patients that underwent a revision TKA after metal LTT sensitivity testing over a 3-year period from January 1, 2015, to December 31, 2017. Patients were referred for metal LTT sensitivity testing after other causes of a painful knee arthroplasty had been excluded (i.e., infection, malalignment, or instability). Patients underwent revision TKA to a hypoallergenic component or a standard component, based on the results of sensitivity testing. Patients testing positive for metal LTT sensitivity were revised to a hypoallergenic component, while those testing negative were revised for arthrofibrosis. Following revision, patients returned to the clinic at an interval of 6 weeks, 5 months, and 12 months for clinical assessment. Patients were excluded if they had incomplete pre-revision data sets. Results from metal LTT sensitivity testing, functional outcomes, pain scores, and satisfaction were analyzed.

### Metal LTT sensitivity testing

Patients were referred for metal LTT sensitivity testing after all other causes of a painful knee arthroplasty had been excluded, including infection, malalignment, or instability via laboratory, radiographic, or mechanical testing, respectively. For sensitivity testing, venous blood was drawn in the out-patient clinic and sent to a third-party laboratory (Orthopedic Analysis, Chicago, IL). Lymphocytes were isolated and incubated for 5 days with a panel of metal allergens (Additional file 1: Figure S1). Reactivity was measured by quantifying lymphocyte proliferation using radioactive markers. Lymphocyte reactivity was normalized based on a known stimulant and reported on a stimulation index (SI). Each allergen received a numeric SI score and was categorized as non-reactive (SI < 2), mildly reactive (SI 2–4), reactive (SI 4–8), or highly reactive (SI > 8) (a sample report is provided in Additional file 2: Figure S2) [21]. Reports for each anonymized patient were entered into a database. Maximum allergy SI for each patient was identified.

### Functional testing

Patient function was assessed by physical exam and survey reporting. During the initial evaluation, range of motion (ROM) of the operative knee was measured in flexion, extension, and total ROM. Instability, measured by varus and valgus laxity, was also recorded. Repeat ROM measures were made at each post-operative visit. Patients were also provided a survey prior to revision and at each post-operative visit to assess their function. The primary outcome variable was walking distance, measured as unable to walk, indoors only, less than 5 blocks, 5–10 blocks, and unlimited. Walking distance categories were entered into the database as an ordinal variable.

### Pain testing

Patients were surveyed prior to their revision regarding multiple characteristics of their TKA pain. They were asked how long the pain has persisted, the intensity of pain 6 months ago on a pain intensity numerical rating scale (PI-NRS) (scale 1–10; 10 being the worst), and current intensity of pain (scale 1–10; 10 being the worst). Change in pain was reported by calculating the percent change in PI-NRS for each patient between time points [22]. Graphically, pain scores were reported as the mean of the PI-NRS over time for each sensitivity group. At each post-operative visit, patients were asked their current intensity of pain. They were also asked to characterize their current pain, measured as none, mild or occasional, mild or occasional stairs only, mild or occasional stairs and walking, moderate occasional, moderate continual, or severe. Pain characterization was converted to a numeric scale of 1–7 (1 = none to 7 = severe) and entered into the database as an ordinal variable.

### Patient satisfaction

At each post-operative visit, patients were asked if they were satisfied with the revision and if they would elect to have the surgery again. Satisfaction was measured as extremely satisfied, very satisfied, moderately satisfied, slightly satisfied, not at all satisfied, and it is too early to tell. Satisfaction was converted to a numeric scale of 1–5 (1 = extremely satisfied to 5 = not at all satisfied; it is too early to tell was excluded from analysis) and entered into the database as an ordinal variable. Patient’s willingness to have the surgery again was entered into the database as a binomial variable of yes = 1 or no = 0 (it is too early to tell was excluded from analysis).

### Statistical analysis

The anonymized dataset was grouped by metal sensitivity as a binary independent variable (non-reactive or reactive) and as an ordinal variable (non-reactive, mildly reactive, reactive, or highly reactive). Data were tested for normal distribution and equal variances before analysis. Means and ranges or standard deviations (SD) were calculated for continuous variables (e.g., age). Categorical variables (e.g., sensitivity level) were expressed as number and percentage. For comparing non-reactive to reactive groups, data were analyzed by means of unpaired two-tailed *t* tests for continuous dependent variables and Mann-Whitney *U* tests for ordinal dependent variables. One-way analysis of variance (ANOVA) was used to test for differences between metal LTT sensitivity levels and pain or ROM; Bonferroni post hoc test were used when appropriate. Pearson’s chi-squared test was used to compare the frequency of willingness to repeat the surgery between the non-reactive or reactive groups. Paired *t* tests were applied for within-group comparisons between pre-revision pain and ROM and post-revision follow-ups. Post-operative pain and ROM were normalized as percentages of baseline (pre-operative) measurements and compared between groups using unpaired two-tailed *t* tests. Correlations between metal LTT sensitivity and percent change in pain or pre-revision pain were tested using Pearson’s product-moment correlation (for metal LTT sensitivity score) or Spearman’s rank correlation tests (for metal LTT sensitivity level). Data was aggregated and analyzed using open-source R [23]. Statistical significance was set at *p* ≤ .05.

## Results

Of the 65 patients identified for the study, 70.8% (46/65) underwent revision TKA and met the criteria for inclusion. Of the patients who underwent revision TKA, 78.3% (39/46) were positive for metal LTT sensitivity (reactive). Both groups had similar pre-revision demographics, function, and pain levels (Table 1). The average age at the time of revision was 61.47 ± 9.34 years. Missing patient data (15.2%; 7/46) was assumed to be at random for the 6-week follow-up dataset and would not introduce bias. For 5- and 6-month datasets, missing data contributed to a significant percentage of the non-reactive sensitivity group and could potentially introduce bias; therefore, no statistical analysis was conducted to compare outcome measures between groups.

**Table 1:** Demographic and outcome data

	All patients	Reactive	Non-reactive	Significance (*p*)
Pre-revision variables	(*n* = 46)	(*n* = 39)	(*n* = 7)	–
Age (years; mean ± SD)	61.47 ± 9.34	61.36 ± 9.98	62.19 ± 2.84	0.704
Female	59%	67%	43%	0.438
Pain duration (months)	8.59 ± 11.73	8.12 ± 11.57	13.67 ± 14.84	0.587
Pain 6 months prior (1–10)	6.59 ± 2.66	6.82 ± 2.55	4.75 ± 3.3	0.303
Pain at revision (1–10)	6.93 ± 2.19	7.15 ± 2.06	5.71 ± 2.63	0.209
Range of motion (degrees)	94.02 ± 26.58	94.74 ± 27.98	90 ± 17.83	0.569
Post-revision #1 variables (6 weeks)	(*n* = 39)	(*n* = 34)	(*n* = 5)	–
Duration (weeks)	6.82 ± 2.53	6.95 ± 2.66	5.94 ± 1.08	0.153
Pain exp	2.97 ± 1.68	2.94 ± 1.7	3.25 ± 1.71	0.748
Pain	4.46 ± 2.79	4.45 ± 2.69	4.5 ± 3.52	0.972
Range of motion (degrees)	101.89 ± 22.08	100.95 ± 23.29	107 ± 13.87	0.366
Post-revision #2 variables (5 months)	(*n* = 31)	(*n* = 29)	(*n* = 2)	–
Duration (weeks)	21.25 ± 17.19	21.68 ± 17.71	15 ± 1.21	–
Pain exp	3.42 ± 1.61	3.52 ± 1.56	1 ± 0	–
Pain	4.19 ± 2.35	4.45 ± 2.2	0.5 ± 0.71	–
Range of motion (degrees)	105.79 ± 20.63	105.35 ± 21.22	112.5 ± 3.54	–
Post-revision #3 variables (1 year)	(*n* = 20)	(*n* = 18)	(*n* = 2)	–
Duration (weeks)	60.89 ± 24.87	59.53 ± 25.7	73.07 ± 13.84	–
Pain exp	3.2 ± 1.86	3.36 ± 1.82	1 ± 0	–
Pain	3.63 ± 2.84	3.91 ± 2.84	1.25 ± 1.77	–
Range of motion (degrees)	112.09 ± 15.62	112.19 ± 16.01	111 ± 15.56	–

Demographics of patients (*n* = 46) who underwent LTT testing prior to hip arthroplasty. Scores from pre- and post-revision assessments of pain and function are reported. Differences between reactive patients and non-reactive patients were compared using unpaired two-tailed *t* tests or Pearson’s chi-squared test where appropriate

### Metal sensitivity

**Fig. 1:**
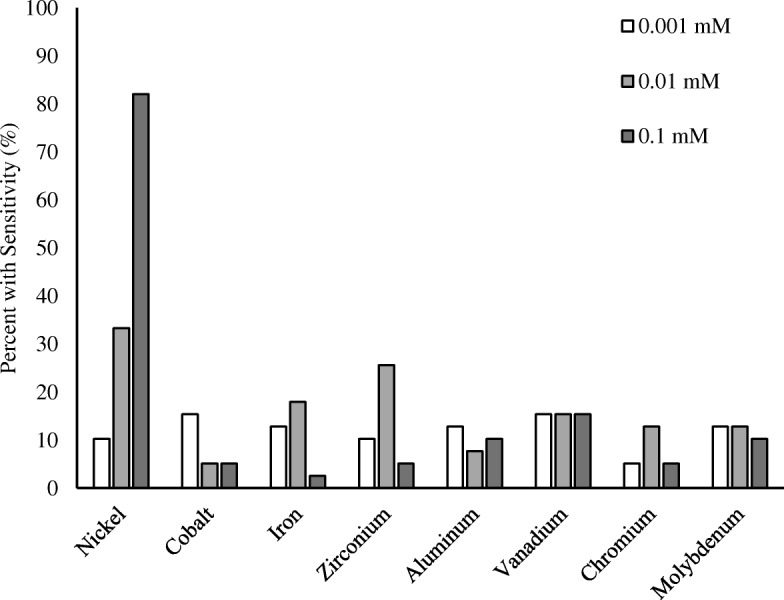
Metal-LTT sensitivity percentage. Percent of all included patients who tested positive for each metal. Metals are subdivided by concentration

**Fig. 2:**
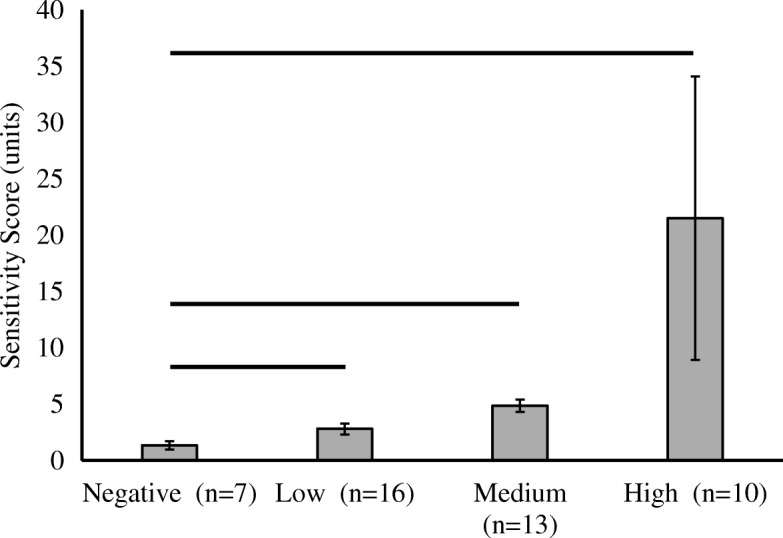
Mean metal-LTT sensitivity score grouped by magnitude of sensitivity. Error bars denote standard deviation and solid horizontal bar denotes a significant (*p* < 0.05) difference in mean compared to the negative sensitivity group

The most common metal sensitivity was to nickel (79.5%, 32/39), and reactivity to other ions is described in Fig. 1. Patients were grouped by their maximum SI across all ions. Only 15.2% (7/46) of patients who underwent revision TKA were non-reactive and tested negative for metal LTT. The average maximum metal sensitivity score of the non-reactive patients was 1.34 ± 0.36. The proportion of the patients testing positive for metal LTT sensitivity was 34.9% (16/46), 28.3% (13/46), and 21.7% (10/46) in the low, medium, and high reactivity groups, respectively. All the patients in the reactive groups had maximum metal sensitivity scores that were significantly higher than the non-reactive group (Fig. 2).

### Function

**Fig. 3:**
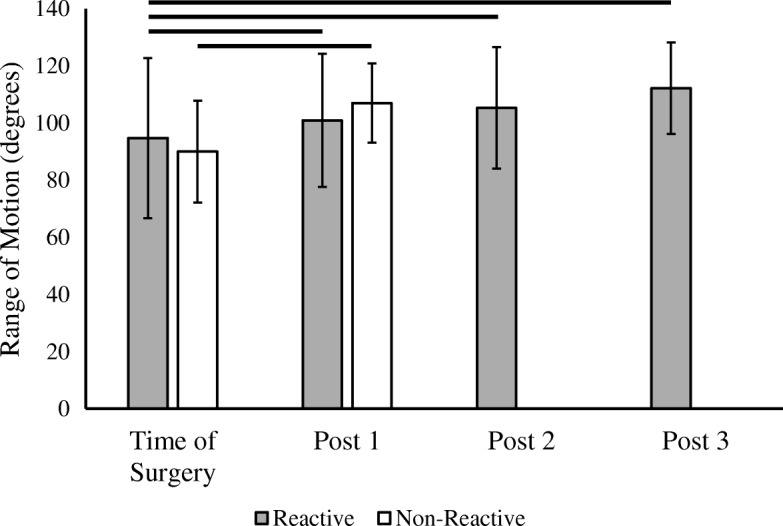
Range of motion in reactive and non-reactive metal-LTT groups. Mean knee flexion/extension range of motion prior to revision and post-revision. There were insufficient non-reactive patients at post-revision 2 and 3 to generate statistical comparisons. Error bars denote standard deviation and solid horizontal bar denotes a significant (*p* < 0.05) difference in mean range of motion between visits

Prior to and 6 weeks post-revision, there was no significant difference in knee flexion/extension ROM between the non-reactive and reactive groups (Fig. 3). However, when compared to baseline measures, both non-reactive and reactive patients significantly improved in range of motion after revision arthroplasty (Fig. 3). There was no significant correlation of metal LTT sensitivity level on ROM pre-revision [F (3) = 1.01, *p* = 0.39] or 6 weeks post-revision [F(3) = 0.31, *p* = 0.82]. However, walking distance ability before revision was significantly higher in the non-reactive group (*W* = 140.5, *p* = 0.044). On average, non-reactive patients could walk 5–10 blocks versus less than 5 blocks in the reactive patients. This significant difference in walking distance persisted at 6 weeks post-revision with the reactive patients maintaining their walking ability of less than 5 blocks, but the non-reactive patients improving to unlimited walking (*W* = 185, *p* = 0.011). While both groups average walking distance improved after revision, the increase was not significant (*p* > 0.05).

### Pain

**Fig. 4:**
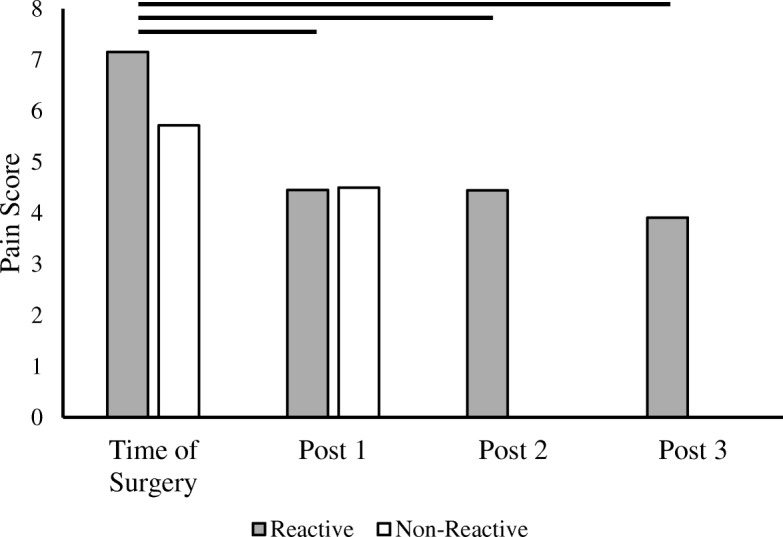
Pain scores in reactive and non-reactive metal-LTT groups. Mean pain score prior to revision and post-revision. There were insufficient non-reactive patients at post-revision 2 and 3 to generate statistical comparisons. Solid horizontal bar denotes a significant (*p* < 0.05) difference in pain score between visits

Intensity of pain was measured at each follow-up visit and compared to the patient’s pre-operative pain level at the time of revision. When comparing metal LTT sensitivity between groups, there was no difference in pain experienced at the time of revision (*p* = 0.21) or at 6 weeks post-revision (*p* = 0.97). However, when comparing metal LTT sensitivity within groups between time points, the reactive group saw a 37.8% decrease in pain at 6 weeks post-revision (*p* < 0.001) (Fig. 4). This reduction in pain continued to improve at 5 months (37.9%; *p* < 0.001) and at 1-year post revision (45.3%; *p* < 0.001). The non-reactive group only saw a moderate, non-significant improvement in pain reduction at 6 weeks post-revision (27.0%; *p* = 0.29). Frequency of pain experienced did not vary significantly between groups at follow-up and did not change significantly between follow-up time points.

**Fig. 5:**
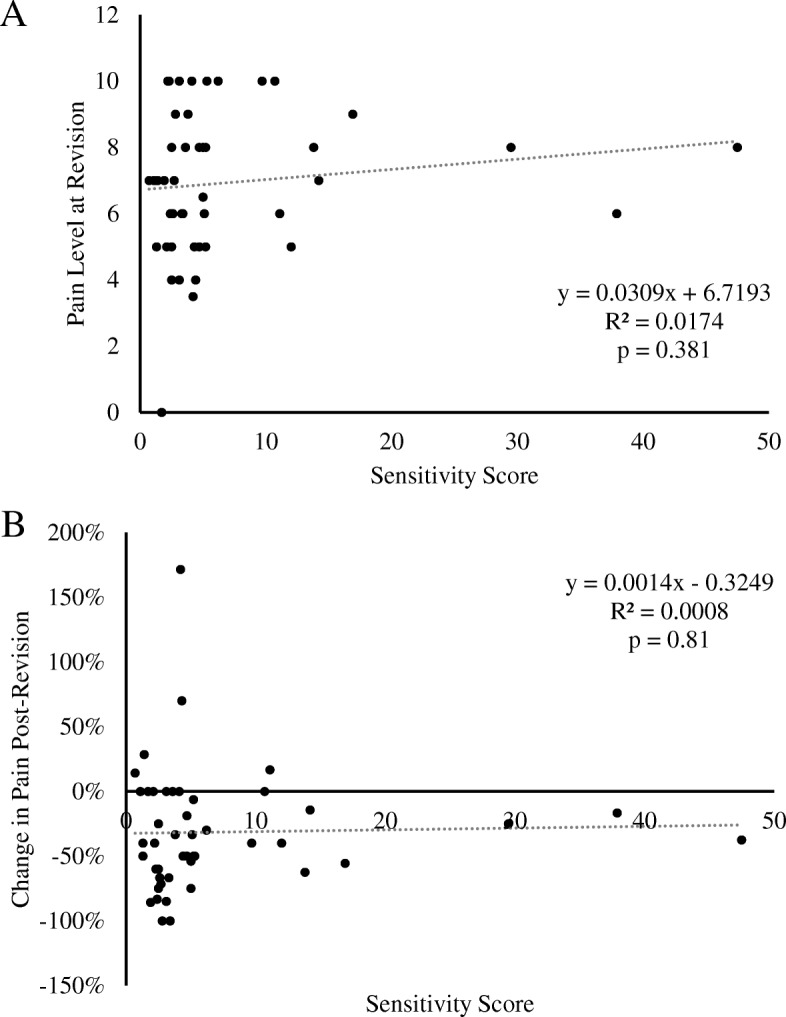
Pain and metal-LTT sensitivity correlation. **a** Correlation between pain level at the time of revision and LTT sensitivity score. **b** Correlation between percent change in pain from pre-revision to post-revision and LTT sensitivity score

Overall, combining patients from both groups, maximum metal LTT sensitivity score did not correlate with pain level at the time of revision (*R*^2^ = 0.02, *p* = 0.38) or percent improvement after revision (R^2^ = 0.001, *p* = 0.81) (Fig. 5a, b). Within the reactive patients, there was a significant effect of metal LTT sensitivity level on pain at 6 weeks post-revision [F (2) = 4.46, *p* = 0.02], but not prior to revision [F(2) = 0.498, *p* = 0.61]. Post hoc comparisons indicate patients with medium LTT sensitivity had a significantly higher pain score than low LTT sensitivity patients (*p* = 0.046). No other comparisons were significant.

### Satisfaction

Only one patient in the cohort reported an unwillingness to undergo the revision arthroplasty again. Overall, all patients reported being very satisfied after revision TKA; there was no difference between positive and negative sensitivity groups (*W* = 62, *p* = 0.89).

## Discussion

Despite overall excellent results following total knee replacements, there remains a subset of patients who are dissatisfied with their outcome. It has been well established that patients with total knee replacements, functional or painful, can test positive for metal sensitivity [7]. However, there is a paucity of studies examining whether a positive metal sensitivity is the cause of the pain or failure and whether revision TKA to a hypoallergenic component would lead to improved results. The purpose of this study was to evaluate whether patients with painful TKA’s testing positive for metal LTT sensitivity have improvement in pain and function once revised to a hypoallergenic component. Furthermore, we sought to compare the pain and functional outcomes of the reactive patients with the outcomes of non-reactive patients following revision.

In this retrospective review, we found a statistically significant decrease in pain following revision TKA to a hypoallergenic implant for patients with a positive metal LTT sensitivity test. Interestingly, patients presenting with a painful TKA, but testing negative for metal sensitivity, did not have a statistically significant improvement in their pain scores post-operatively, indicating that metal sensitivity is likely to be a pain generator (Fig. 4). However, both patients testing negative for metal LTT sensitivity and those testing positive had a significant increase in range of motion (ROM) post-operatively (Fig. 3). Additionally, patients testing positive for metal LTT sensitivity reported significantly lower walking function, as measured by the number of blocks walked, compared to the non-allergy group following revision TKA. Surprisingly, the degree of metal reactivity inversely correlated to pain relief post-operatively following revision TKA. A potential explanation is patients with a lower pre-operative metal reactivity have less allergen and thus a greater degree of pain relief once the allergen is removed. Whereas those with a higher level of reactivity on have a portion of the allergen removed with surgery. Further long-term follow-up studies are needed to determine if the allergen level would continue to decrease over time, resulting in more pain relief as the allergen is cleared by the patients system.

The results of this study are in agreement with several case reports in the literature Caicedo et al. demonstrated a significant percentage of patients that had undergone total joint arthroplasty and had unexplained joint pain were hypersensitive to implant metals [24]. Bao et al. presented a case report in which a patient with continuous swelling of their total knee arthroplasty showed significant improvement after a synovectomy and component revision [25]. Overall, in the absence of obvious etiology, patients presenting with pain, decreased range of motion, and even contact dermatitis-like reactions have resolution of their symptoms after revision to a hypoallergenic component. Recently, Postler et al. published a randomized controlled trial evaluating the outcomes of a coated versus uncoated total knee arthroplasty [26]. However, they excluded all patients with a known metal hypersensitivity and, not surprisingly, showed no difference in patient-reported outcomes. They also looked at metal ion levels after implantation and showed a significant increase in chromium concentrations following standard TKA as opposed to a coated, hypoallergenic TKA. Interestingly, there was no increase in cobalt or nickel, which was the most common allergen in our study. This may have been due to their exclusion of patients with metal hypersensitivities indicating that it is not only the increased level of metal ion but that those with increased metal ions are most susceptible to an increase in that specific ion level. Similar to our results, Yang at al. found no correlation between LTT level and post-revision KSS or range of motion [20]. They also performed histopathologic assessment of the periprosthetic tissue samples and scored them using the aseptic lymphocyte-dominated vasculitis-associated lesion (ALVAL) scoring system. There was no correlation between LTT level and the ALVAL score suggesting a positive LTT alone may not be sufficient to diagnose TKA failure.

There are several limitations to our study. As with any retrospective review, there is inherent bias associated with the study. All revisions were performed by a fellowship-trained total joint surgeon, specializing in revision arthroplasty at a single institution, which introduces possible selection bias. While all revision surgeries were performed by a single surgeon, the primary arthroplasties were performed by numerous surgeons across a vast geographic area. Of the patients included in this retrospective review, 15.2% were lost to follow-up at the 6-week time point. While this could potentially introduce bias, there was proportional loss between reactive and non-reactive groups and total loss was below the suggested threshold of > 20% at which the threat to validity is increased [27]. Comparison of outcomes between reactive and non-reactive groups was limited to 6 weeks due to insufficient patients in the non-reactive group at the 5-month and 12-month time points for statistical analysis. While the focus of this retrospective review was to report the functional outcome of reactive patients who underwent revision, comparisons to non-reactive patients was a secondary aim. The only significant difference between the reactive and non-reactive groups at 6 weeks was a moderate increase in functional walking distance in the non-reactive group which was also seen pre-revision. Our data show that the walking distance of reactive patients at the 5-month and 12-month time points improved to the functional level of non-reactive reactive patients at the 6-week time point. However, a larger sample size of non-reactive patients at the 5-month and 12-month time points would be needed for direct comparison. Lastly, the reliance on patient-reported outcomes for our data may have introduced bias in our results. With these limitations taken into account, the authors believe the results are valuable as the volume of clinical data on outcomes after revision to a hypoallergenic TKA in patients with metal sensitivities is limited.

## Conclusion

To the best of our knowledge, this is the largest reported study on pain and functional outcomes for patients testing positive and revised for metal sensitivity. In this study, we found that patients presenting with a painful knee arthroplasty and positive metal LTT sensitivity have improved pain scores, walking function, and range of motion following revision to a hypoallergenic component. Lastly, this study also provides a treatment approach for patients presenting with a painful knee replacement, in order to provide a timely diagnosis and effective management. The results of this study have changed the senior authors’ clinical practice in two ways. First, the workup of a patient with a painful knee arthroplasty now includes metal-LTT after infection, malalignment, and instability have been ruled out. If the patient tests positive, a revision to a hypoallergenic component is recommended. Additionally, if a patient with a known metal sensitivity presents for a primary total knee arthroplasty, the patient is treated with a hypoallergenic component, without pre-testing, in order to avoid future complications of metal hypersensitivity and the morbidity of subsequent revision surgeries.

In summary, it is encouraging that patients with a painful TKA and positive LTT metal sensitivity may benefit from revision TKA to a hypoallergenic component. This can help to capture a subset of patients dissatisfied with their surgeries and provide a solution for their unexplained pain. This study also provides a potential treatment algorithm for patients presenting with a painful knee replacement, in order to provide effective and timely diagnosis and management. Additionally, pre-testing patients with a known metal sensitivity could detect patients who are at risk for developing a reaction to a standard implant and avoid future revision surgeries by undergoing primary TKA with a hypoallergenic component. Long-term, prospective multi-center studies are needed to further evaluate the results in the future.

## Additional files

Additional file 1: **Figure S1.** Metal allergens panel. (XLSX 10 kb)

Additional file 2: **Figure S2.** Sample metal-LTT sensitivity report. Provided by Orthopedic Analysis, Chicago, IL [21]. (PDF 210 kb)

## Data Availability

The datasets used and/or analyzed during the current study are available from the corresponding author on reasonable request.

## Abbreviations

ALVAL: Aseptic lymphocyte-dominated vasculitis-associated lesion
ANOVA: Analysis of variance
HIPPA: Health Insurance Portability and Accountability Act
IRB: Institutional review board
LTT: Lymphocyte transformation test
ROM: Range of motion
SD: Standard deviations
SI: Stimulation index
TKA: Total knee arthroplasty
