# Supplementary material for: Improved outcomes in patients with positive metal sensitivity following revision total knee arthroplasty
Source: J Orthop Surg Res. 2019 Jun 17;14:182. doi: 10.1186/s13018-019-1228-4 (PMC6580588; doi:10.1186/s13018-019-1228-4)
Supplement: Supplementary file 2 — Figure S2. Sample metal-LTT sensitivity report. Provided by Orthopedic Analysis, Chicago, IL [21]. (PDF 210 kb) [file 13018_2019_1228_MOESM2_ESM.pdf]

Metal-LTT Analysis Report

Panel 1

**Report Date**	1/1/2011		
**Report Time**	12:00 AM	**Sample Collected**	1/1/2011
**Patient ID**	1234	**Sample Received**	1/1/2011 10:00 AM
**Report For**	**Doe, John**	**DOB**	xx/xx/xx
**Attending Physician**	**Dr. X**		

**Control cpm**	3122.0
**Positive control (PHA) cpm**	30358.3

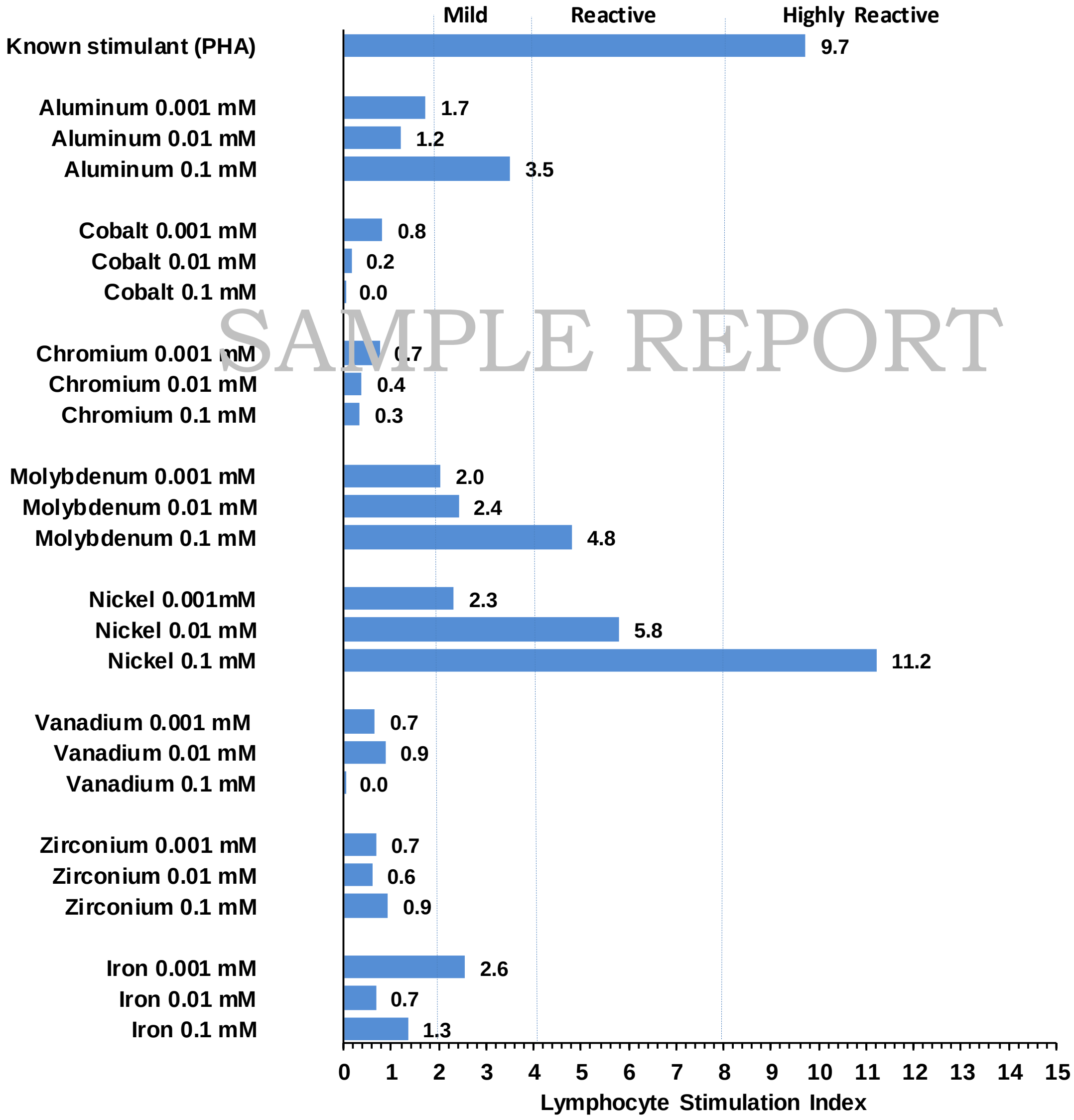

SAMPLE REPORT

Mildly Reactive	2 to 4
Reactive	4 to 8
Highly Reactive	above 8

ORTHOPEDIC ANALYSIS

**Report Date**	1/1/2011		
**Report Time**	12:00 AM	**Sample Collected**	1/1/2011
**Patient ID**	1234	**Sample Received**	1/1/2011 10:00 AM
**Report For**	**Doe, John**	**DOB**	xx/xx/xx
**Attending Physician**	**Dr. X**		
Control cpm	3122.0		
Positive control (PHA) cpm	30358.3		

Metal Challenge	Stimulation Index	Range (percentile based)
Known Stimulant (PHA)	9.7	Internal Control Passed
Aluminum	3.5	**Mildly Reactive**
Cobalt	0.8	
Chromium	0.7	
Molybdenum	4.8	**Reactive**
Nickel	11.2	**Highly Reactive**
Vanadium	0.9	
Zirconium	0.9	
Iron	2.6	**Mildly Reactive**

SAMPLE REPORT

IMPORTANT DISCLAIMER

Metal-LTT is a highly quantitative blood assay that has been used in many published scientific studies of metal allergy. This testing is performed per-customer request . The results of this testing are the property of the customer and should be used in combination with patient evaluation for diagnosis. It remains unclear if metal hypersensitivity in general is etiologically linked to poor implant performance.
